# Micro-Costing Analysis for the Treatment of Atrial Fibrillation: An Economic Evaluation of the First Italian Experience of Same-Day Discharge Protocol for Cryoballoon Atrial Fibrillation Ablation

**DOI:** 10.3390/jcm13102836

**Published:** 2024-05-11

**Authors:** Giuseppe Sgarito, Antonio Cascino, Giuliano Ferrara, Sergio Conti

**Affiliations:** Department of Electrophysiology, ARNAS Civico-Di Cristina-Benfratelli, 90127 Palermo, Italy; giuseppe.sgarito@arnascivico.it (G.S.); antonio.cascino@arnascivico.it (A.C.); giuliano.ferrara@arnascivico.it (G.F.)

**Keywords:** atrial fibrillation, catheter ablation, cryoballoon ablation, same-day discharge, economic analysis

## Abstract

**Background**: Atrial fibrillation (AF) is the most common cardiac arrhythmia, and its prevalence is expected to increase further due to the aging population, increasing prevalence of risk factors, improving detection methods, and broadening of catheter ablation indications. Along with limited healthcare resources and bed availability, these reasons led to the development of a same-day discharge (SDD) protocol. The aim of this study was to evaluate the health and economic impact of a routine adoption of same-day discharge after cryoballoon AF ablation. **Methods**: Consecutive patients with symptomatic and drug-refractory AF scheduled for first-time AF ablation were screened, and if deemed suitable, the SDD protocol was proposed and, if accepted, enrolled in the protocol. **Results**: A total of 324 patients were screened, and 118 were considered eligible for the SDD pathway. Fifty-two patients accepted the SDD pathway and were included in this study. The analysis showed that the variation in resource consumption associated with cryoablation in SDD is equal to EUR 739.85/patient. The analysis showed that the main cost driver for ordinary hospitalization was represented by the hospital stay, which was calculated to be 36% of the total cost. In total, there was a cost reduction of EUR 38.472 thanks to optimized AF patient management from the standard recovery setting to SDD. **Conclusions**: SDD after cryoballoon ablation of AF is feasible in selected patients with a standardized protocol.

## 1. Introduction

Atrial fibrillation (AF) is the most common cardiac rhythm disorder. AF is associated with increased morbidity and mortality, resulting in a high burden on the healthcare system. AF prevalence is estimated to grow further in the following decades. Projection studies show that the prevalence of AF will rise to 15.9 million in 2050 in America and 17.9 million in 2060 in Europe [1]. Catheter ablation (CA) is a well-established treatment for atrial fibrillation (AF) with superior long-term success rates compared to antiarrhythmic drug therapy alone for both paroxysmal AF (PAF) and persistent AF (Pe-AF) [2,3]. Recently, the EAST-AFNET 4 Trial showed that early rhythm-control therapy was associated with a lower risk of adverse cardiovascular outcomes than usual care among patients with early atrial fibrillation and cardiovascular conditions [4]. Due to the aging population, increasing prevalence of AF risk factors, improving detection methods for AF, and broadening indications for ablation, in many centers, CA for AF is the most common resource-consuming ablation procedure performed, strongly impacting waiting list times and healthcare costs [5]. The combination of these factors and limited bed availability led to the development of a same-day discharge (SDD) protocol in some centers worldwide with acceptable efficacy and safety outcomes [6,7], and data supporting this approach are proliferating. SDD has already been shown to be safe and effective for non-complex ablations [8], cardiac implantable electronic device procedures, and left atrial appendage occlusion. However, CA of AF is a longer and more complex procedure, requiring deep sedation or general anesthesia and high levels of procedural anticoagulation; for those reasons, AF ablation has traditionally involved at least one overnight stay in the hospital. Moreover, to obtain full reimbursement in Italy, patients must spend two nights in the hospital. Due to the concurrent COVID-19 outbreak, we started the SDD pilot project in our institution to reduce patient hospital stays and time spent on the waiting list. Our objective was to assess our day-case service’s overall effectiveness and safety. Furthermore, we aimed to evaluate the health and economic impact of a routine adoption of day-case AF ablation.

## 2. Materials and Methods

All consecutive patients with symptomatic and drug-refractory PAF or early Pe-AF scheduled for de-novo catheter ablation of AF at the Electrophysiology Department of ARNAS Civico Hospital were screened between September 2020 and September 2022. If deemed suitable, the SDD protocol was proposed to the patient, and in case of acceptance, they were prospectively enrolled in this study. PAF and Pe-AF were defined according to the latest guidelines [9]. Baseline patients’ clinical characteristics were comprehensively reviewed from the medical records, including comorbidities and the mean AF duration. This study complied with the Declaration of Helsinki, and all patients gave written informed consent before the procedure. This protocol was offered to the patients as a quality improvement pilot project.

**Figure 1:**
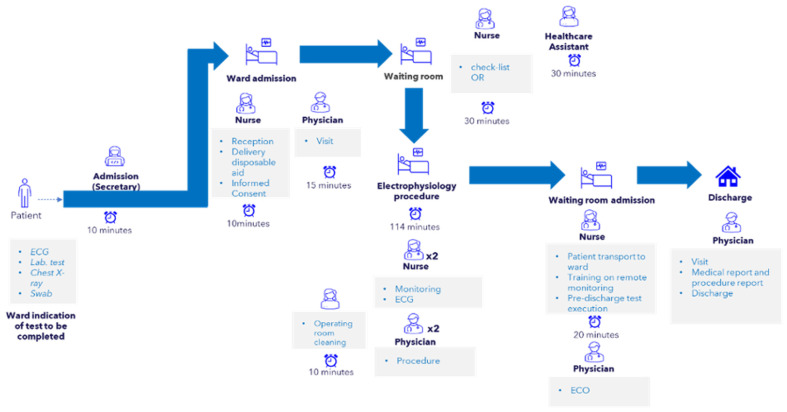
Same-day discharge workflow from patient admission to discharge.

The economic analysis was conducted using the activity-based costing method and it was conducted assuming an ordinary hospitalization versus an SDD in the same group of patients. The different phases of cryoablation treatment for atrial fibrillation have been examined to define resource consumption and the time healthcare staff dedicated to each stage (Figure 1). The analysis evaluated the costs associated with each phase, allowing the definition of the total costs for each hospital pathway (ordinary admission—OR, overnight day-surgery—ODS, day-surgery—SDD). Data on patient pathway costs and dedicated staff time have been collected through clinical and administrative staff interviews. Unit costs of drugs, imaging, laboratory tests, disposables, medical procedures, and visits were sourced from the hospital’s administration, while non-clinical staff and overhead were quantified through literature (discounted costs). Personnel costs were computed based on a per-minute basis.

### 2.1. Inclusion Criteria

Patients were routinely seen in a dedicated physician-led preassessment clinic 1–2 weeks before the planned ablation date; all first-procedure AF ablations were considered for SDD, and suitability for day-case was based on several factors including patient age, frailty, and comorbidities, distance traveled, patient wishes, and appropriate social support on discharge. Patients were included in this study if they had the following clinical and non-clinical criteria for an SDD protocol, as defined by Rajendra et al. [10], and summarized in Table 1:

- Clinical factors: (a) stable anticoagulation; (b) no history of bleeding; (c) no systolic heart failure; (d) no history of pulmonary disease; (e) no interventional procedure within 60 days from catheter ablation; (f) body mass index (BMI) <35; (g) CHA2DS2-VASc ≤ 3; (h) non-severely dilated left atrium; (i) age < 65 years old; (j) suitable candidate for cryoballoon ablation.
- Non-clinical factors: (a) home residence within 50 km, or if more, with an emergency department (ED) reachable within 30 min; (b) home assistance to the patients the same day of the procedure.

### 2.2. Exclusion Criteria

Patients were excluded if they met the following exclusion criteria: (a) they were unwilling or unable to consent; (b) in case of the presence of any contraindications to AF ablation; (c) pregnancy or breastfeeding; (d) comorbidities with life expectancy <1 year; (e) contraindications to oral anticoagulation therapy; (f) unwilling to consent to SDD protocol; (g) concomitant atrial flutter / atrial tachycardia requiring mapping.

### 2.3. Hospital Admission and Ablation Procedure

SDD workflow is illustrated in Figure 1. Patients who were deemed eligible for and accepted an SDD procedure were admitted to a cardiac short-stay unit on the day of ablation at 7 am. A 12-lead ECG and blood sample examination were performed at arrival. In the case of a CHA2DS2-VASC score ≥2 and AF recorded in the morning, transesophageal echocardiography (TEE) was scheduled before admission to the electrophysiology lab. The periprocedural anticoagulation strategy was uninterrupted or minimally interrupted [11]. The catheter ablation procedure was performed with the 28 mm Arctic Front Advance Pro^TM^ cryoballoon ablation catheter (Medtronic, Medtronic, Inc., Minneapolis, MN, USA) as the first case of the day (usually two cryoballoon cases per day). No preprocedural imaging with either magnetic resonance or computed tomography was performed. All patients were treated according to standard clinical practice and were ablated by one or two experienced operators beyond the learning curve. All procedures were carried out in conscious sedation using midazolam, fentanyl, and dexmedetomidine infusion without the presence of an anesthesiologist, available on call, but with the presence of a second physician in the electrophysiology lab. The doses of midazolam, fentanyl, and dexmedetomidine were based on previously published data [12]. All lab staff were trained in managing cardiac sedation and advanced cardiac life support. A 6F deflectable decapolar catheter was inserted through the right femoral vein and placed into the coronary sinus to guide the transseptal puncture and stimulate the right phrenic nerve while treating the right pulmonary veins (PVs). Ultrasound-guided femoral vein access was at the operator’s disposal and discretion but not routinely used in our series. A single transseptal puncture was performed using a needle system (BRK XS, Abbott Medical, Minneapolis, MN, USA) and a standard transseptal sheath (SL0 8F or 8.5F, Abbott Medical, Minneapolis, MN, USA), subsequently exchanged with a steerable 15F sheath (FlexCath, 15F, Medtronic, Inc., Minneapolis, MN, USA). After transseptal puncture, heparin was administered intravenously as a bolus, followed by a continuous infusion (1000 U/h) reaching ACT level >300 s. The FlexCath was continuously irrigated with heparinized saline (2 mL/h). An esophageal temperature probe was used in all patients (Esotherm Plus, FIAB) to monitor intraesophageal temperature decrease and adjusted during the procedure to stay as close as possible to the ablation catheter. Cryotherapy was interrupted if the endoluminal esophageal temperature dropped below 18 °C. One cryotherapy application per pulmonary vein (PV) was delivered, 180–240 s each, aiming for a minimum temperature of less than −40 °C. After treatment of all PVs, the entrance block was confirmed with high-output pacing (12 V @ 2.9 ms) using the Achieve mapping catheter (Medtronic, Inc., Minneapolis, MN, USA). “Far-field” capture and sensing were ruled out using differential pacing maneuvers. Any residual conduction into the PVs was treated by further cryotherapy applications. At the procedure’s end, protamine was given to reverse the heparin if ACT > 350 s, and the two femoral venous sheaths were removed in the electrophysiology laboratory. Hemostasis was obtained through a figure-of-eight (FOE) suture and manual pressure. No arterial sheaths were placed. Urinary catheters were not routinely placed. An Implantable Loop Recorder (ILR) was offered to all patients.

If the procedure was completed by 2 pm, the patient was admitted for observation in the recovery area without inpatient bed utilization for at least 6 h. Then, after evaluation by an allied professional (AP), if vital signs were stable (systolic BP > 100 mmHg, SO_2_ > 95% on room air, normal mental status), no recurrence of arrhythmia occurred, no evidence of hematoma or bleeding after removing purse stitches, and ambulation was without difficulty, the patient was discharged home after adequate education. Postprocedural echocardiography was routinely used before discharge to rule out pericardial effusion. The attending physician made the final decision, depending on the case details and according to patient preference. If needed, overnight observation in the inpatient ward was at their disposal. If a procedure was completed after 2 pm, it was recognized as ODS, and the patient was admitted into the cardiology ward.

### 2.4. Follow-Up

All patients included in this study were evaluated in the outpatient clinic at 3, 6, and 12 months. At each visit, a standard 12-lead ECG was recorded. Oral anticoagulants were continued eight weeks after ablation and then managed according to the CHA2DS2-VASc. AADs were withdrawn at three months or continued at the physician’s discretion. Moreover, data recorded from the ILRs were remotely and on-site collected to evaluate the occurrence of atrial tachycardia (AT), atrial flutter (AFL), and AF episodes. Each follow-up focused on the assessment of atrial arrhythmia-related symptoms and AF burden. Atrial arrhythmia recurrence was defined as any documented AT, AFL, and AF episode lasting longer than 30 s. The AF burden was calculated as the percentage of time in AF between each follow-up visit based on manually adjudicated episodes. Any arrhythmia observed within three months after ablation was defined as early AF and not considered an arrhythmia recurrence.

### 2.5. Statistical Analysis

This was a single-center prospective study. All clinical characteristics are reported as descriptive statistics. Continuous variables are expressed as mean ± standard deviation. Categorical variables were reported as percentages. A *p*-value of <0.05 was considered statistically significant. Freedom from arrhythmia was generated by the Kaplan–Meier method. All statistical tests were performed using SPSS for Windows 25.0 (SPSS, Chicago, IL, USA).

## 3. Results

### 3.1. Patient Population and Procedure Characteristics

Out of 324 patients screened, 118 patients were deemed eligible for the SDD pathway. Finally, fifty-two patients accepted the SDD pathway and were included in the study protocol. All patients underwent pulmonary vein isolation (PVI) with the Arctic Front Advance Pro^TM^ cryoballoon ablation catheter. The baseline clinical characteristics are reported in Table 2. All the included patients had symptomatic (EHRA IIb, III, and IV) and drug-refractory AF. The procedural characteristics are reported in Table 3. The median value and interquartile range for the total procedure and fluoroscopy time were 113 (65–122) and 25 (15.0–30.0) minutes, respectively. In the overall population, no acute procedural complications occurred. Among the patients included in this study, 46% had an ILR implanted (*n* = 24). The mean follow-up time was 15.6 ± 9 months. During the follow-up period, nine patients (17.3%) had at least an episode of atrial arrhythmias recurrence, while considering the blanking period, 11/52 (21%) patients had at least one detected AF episode. The annual rate of AF recurrences was 9.72.

### 3.2. Economic Analysis

**Figure 2:**
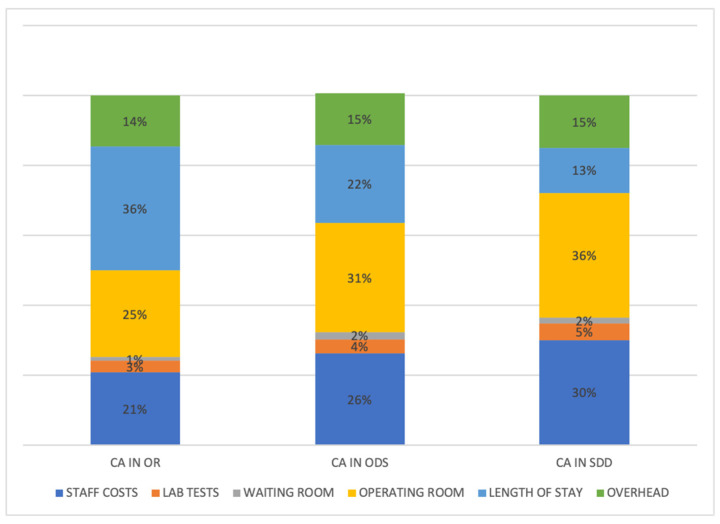
Distribution of costs related to different patients’ pathways for catheter ablation (CA) (ordinary admission—OR, overnight day-surgery—ODS, day-surgery—SDD). Please note it is the same group of patients.

The economic analysis showed that the resource consumption associated with the execution of cryoballoon ablation of AF under ordinary hospitalization (two nights of hospitalization in the ward of the cardiology department) was equal to EUR 2415.12 compared to EUR 1921.89, which is, instead, the cost of the day-surgery overnight pathway. The costs associated with the SDD patient pathway were found to be equal to EUR 1.675 The focus was on the costs of the patient journey and not on devices, drugs, and consumables that were equally used in all three care pathways. The main cost driver for the path in ordinary hospitalization was represented by the hospital stay, which represented 36% of the total, differently from ODS and SDD pathways, which represented 22% and 13% of the total, respectively (Figure 2). The analysis showed that the variation in resource consumption associated with the execution of cryoablation in the OR compared to SDD is equal to EUR 739.85 per patient. The comparison between the OR and ODS pathways is equal to EUR 493.24. The data collected were analyzed to identify areas where efficiency can be improved or costs reduced. All 52 patients included were treated following the SDD pathway. From a hospital perspective, we saved EUR 38.472 thanks to optimized AF patient management from a standard recovery setting to same-day discharge.

## 4. Discussion

The safety and feasibility of SDD for patients undergoing catheter ablation for less complex arrhythmias have been previously described in the published literature [8]. Currently, there is no consensus about an SDD protocol to be applied for AF ablation, and the most recent guidelines do not provide any recommendations about early discharge after this procedure. The main reason for the routine admission of patients undergoing catheter ablation of AF is related to the complexity of the procedure and the periprocedural risks. Historically, AF ablation has been perceived as a long and complex procedure, often requiring general anesthesia and an overnight stay. The severe procedure-related complication rate ranges from 2.44% to 6.29% and depends on the volume of procedures regularly performed by the center, techniques and strategies applied, and clinical management of the patients following AF ablation [9,13,14,15].

Moreover, among the factors implicated in implementing an SDD protocol into clinical practice is the physician’s perception of SDD. In a recent survey performed within the European electrophysiologists’ community, less than 20% stated that SDD was implemented in their institution following AF ablation, and yet, about half of the physicians could not imagine including SDD protocols in their workflow in the future [16]. On the other hand, Aguilera et al. reported higher patient satisfaction in an SDD group of patients compared to admitted patients [17]. Lately, there has been an increased interest in SDD after ablation procedures, not only because of the COVID-19 pandemic but also due to a more conscious healthcare utilization. Moreover, longer and unjustified in-hospital stays can increase the risk of hospital-acquired complications, which are potentially reduced with the SDD pathway [18].

Growing evidence shows that SDD following catheter ablation of AF is feasible, safe, and cost-saving [19,20,21,22]. Although the selection and discharge criteria vary by study, the success rate of SDD has been suggested to be between 79.1% and 99.2% among patients who meet the requirements for SDD. Recently, Rajendra et al. published the largest US experience of the SDD protocol. In this prospective multicenter study, the primary efficacy endpoint—success rate of SDD—was achieved in 86.1% of patients. The readmission rate was similar between patients in the SDD and non-SDD groups. Interestingly, patients in the SDD group had lower acute complications when compared to the non-SDD group. The SDD protocol did not have an impact in terms of freedom from any atrial arrhythmia during follow-up [23].

As far as we know, this is the first Italian and among the first European experiences reporting an SDD protocol applied to catheter ablation of AF using cryoenergy. In our study, SDD after cryoballoon ablation of AF is feasible, and this approach was not associated with significant hospital readmission or complication rates after discharge. In addition, the micro-costing analysis demonstrated the benefit of SDD compared to ordinary hospitalization regarding efficiency and economic sustainability. Economic savings have been generated due to the reduction in costs associated with hospital stays (such as patients’ overnight stays and the use of hospital beds, meals, and related services). Wider adoption of day-surgery cryoablation AF procedures, following careful patient selection, would enhance resource consumption optimization and lead to lower patient management costs.

In conclusion, the analysis gave us a clear overview of the potential positive impact of switching AF cryoablation procedures to SDD management in selected patients. Our regional healthcare system has day-surgery tariffs that allow us to perform some procedures in the right setting and with appropriate reimbursement. It would be desirable to follow the virtuous example of the Sicily region, which has adopted an ad hoc reimbursement for day-surgery patient management, as it could help ensure the economic sustainability of the Italian healthcare system while maintaining high quality and safety standards for the patient. Ad hoc tariffs not only allow the correct implementation of technological innovation but also enable the execution of procedures in settings that lower the consumption of resources. Prospective and larger studies are needed to confirm results for contemporary ablation techniques, structural obstacles for further SDD implementation must be tackled, and data regarding patients’ perceptions of SDD in the context of AF ablation are required. Ultimately, SDD protocols could be a promising approach to overcoming the increasing demands on interventional electrophysiology.

## 5. Limitations

Our study has several limitations. First, it is a single-center, prospective study with a small number of very well-selected patients. Roughly one-third of patients screened were deemed eligible for an SDD pathway. Among the selected patients, 52 accepted to be included in the study protocol. However, the relatively low number of patients that accepted the SDD pathway can be explained since this was a pilot project and an SDD protocol for AF ablation has never been implemented in our regional healthcare system. Second, due to a rigorous selection, most patients were young and had few comorbidities. As such, the results of this study may not be generalizable to patients with comorbidities and those who are older.

## 6. Conclusion

SDD after cryoballoon ablation of AF is feasible and cost-effective in selected patients with a standardized protocol. The clinical efficacy and economic sustainability of the procedure are guaranteed even in an inpatient SDD pattern with less resource consumption, highlighting advantages for the hospital such as reduction in patient management costs, shortening the length of stay, and freeing up of beds (allowing them to be reallocated where needed). Another meaningful impact of a routine adoption of day-surgery AF ablation is the reduction in waiting lists, accelerating patient access to care by optimizing resource consumption. With an appropriate reimbursement, SDD also becomes economically viable from a regional perspective. Further and randomized data are needed to widen the number of patients with an indication for SDD.

## Figures and Tables

**Table 1:** Inclusion and exclusion criteria.

Inclusion Criteria	Exclusion Criteria
(1) Clinical factors	(1) Clinical factors
- Stable anticoagulation	- Unwilling or unable to consent
- No history of bleeding	- Contraindications to AF ablation
- No systolic heart failure	- Pregnancy or breastfeeding
- No history of pulmonary disease	- Life expectancy <1 year
- BMI < 35	- Contraindications to OAC
- CHA2DS2-VASc ≤ 3	- Concomitant AT/AFL requiring mapping
- Non-severely dilated left atrium	
- Age < 65 years old	
- Suitable for cryoballoon ablation	
(2) Non-clinical factors	(2) Non-clinical factors
- Home residence within 50 km	- Unwilling to consent to SDD protocol
- ED reachable within 30 min	
- Home assistance to the patients the same day of the procedure	

BMI = body mass index; ED = emergency department; AF = atrial fibrillation; OAC = oral anticoagulation; AT = atrial tachycardia; AFL = atrial flutter; SDD = same-day discharge.

**Table 2:** Baseline clinical characteristics of patient population.

Baseline Characteristics	TOTAL (*n* = 52)
Age at first ablation (years), mean ± SD	57.3 ± 9
Gender (Female), % (*n*)	28.8% (15)
Body Mass Index (Kg/m^2^), mean ± SD	27.2 ± 4.4
Type of Atrial Fibrillation	
Paroxysmal, % (*n*)	96.1% (50)
Persistent, % (*n*)	3.9% (2)
Months from AF diagnosis to PVI (median I–III IQR)	30.0 (12.0–48.0)
Previous tested AADs ≥2, % (*n*)	65.3% (34)
Mean EHRA class, mean ± SD	2.8 ± 0.6
History of Stroke/TIA, % (*n*)	1.9% (1)
Hypertension, % (*n*)	51.9% (27)
Mean CHA_2_DS_2_-VASc, mean ± SD	1.8 ± 1.2
Diabetes, % (*n*)	7.6% (4)
Chronic Kidney Disease, % (*n*)	1.9% (1)
Left Ventricular EF %, mean ± SD	58.4 ± 4.7
LA Diameter, mm (median I–III IQR)	41.3 (38.0–44.0)
Class I or III AADs, % (*n*)	90.4% (47)
OAC % (*n*) Distance home–Hospital in km, mean ± SD	100% (52) 23.8 ± 1.4

AF = atrial fibrillation; AADs = antiarrhythmic drugs; PVI = pulmonary vein isolation; TIA = transient ischemic attack; LVEF = left ventricular ejection fraction; LA = left atrium; CV = cardiovascular.

**Table 3:** Procedure characteristics.

Procedure Characteristics	
Procedure duration, mean ± SD (min)	83 ± 32
Fluoroscopy duration, mean ± SD (min)	15.1 ± 11
Catheter ablation time, mean ± SD (min)	18.2 ± 9
Left atrium dwell time, mean ± SD (min)	34.2 ± 12
Acute success rate (n. treated veins/n. target veins), %	100%

## Data Availability

Data are available upon reasonable request.
